# tigaR: integrative significance analysis of temporal differential gene expression induced by genomic abnormalities

**DOI:** 10.1186/1471-2105-15-327

**Published:** 2014-10-02

**Authors:** Viktorian Miok, Saskia M Wilting, Mark A van de Wiel, Annelieke Jaspers, Paula I van Noort, Ruud H Brakenhoff, Peter JF Snijders, Renske DM Steenbergen, Wessel N van Wieringen

**Affiliations:** Department of Epidemiology and Biostatistics, VU University Medical Center, P.O. Box 7057, 1007 MB Amsterdam, The Netherlands; Department of Pathology, VU University Medical Center, P.O. Box 7057, 1007 MB Amsterdam, The Netherlands; Department of Mathematics, VU University Amsterdam, De Boelelaan 1081a, 1081 HV Amsterdam, The Netherlands; InteRNA Technologies BV, Padualaan 8, 3584 CH Utrecht, The Netherlands; Department of Otolaryngology/Head-Neck Surgery, VU University Medical Center, P.O. Box 7057, 1007 MB Amsterdam, The Netherlands

**Keywords:** Integration, Empirical Bayes, Semi-parametric, INLA, High-dimensional

## Abstract

**Background:**

To determine which changes in the host cell genome are crucial for cervical carcinogenesis, a longitudinal *in vitro* model system of HPV-transformed keratinocytes was profiled in a genome-wide manner. Four cell lines affected with either HPV16 or HPV18 were assayed at 8 sequential time points for gene expression (mRNA) and gene copy number (DNA) using high-resolution microarrays. Available methods for temporal differential expression analysis are not designed for integrative genomic studies.

**Results:**

Here, we present a method that allows for the identification of differential gene expression associated with DNA copy number changes over time. The temporal variation in gene expression is described by a generalized linear mixed model employing low-rank thin-plate splines. Model parameters are estimated with an empirical Bayes procedure, which exploits integrated nested Laplace approximation for fast computation. Iteratively, posteriors of hyperparameters and model parameters are estimated. The empirical Bayes procedure shrinks multiple dispersion-related parameters. Shrinkage leads to more stable estimates of the model parameters, better control of false positives and improvement of reproducibility. In addition, to make estimates of the DNA copy number more stable, model parameters are also estimated in a multivariate way using triplets of features, imposing a spatial prior for the copy number effect.

**Conclusion:**

With the proposed method for analysis of time-course multilevel molecular data, more profound insight may be gained through the identification of temporal differential expression induced by DNA copy number abnormalities. In particular, in the analysis of an integrative oncogenomics study with a time-course set-up our method finds genes previously reported to be involved in cervical carcinogenesis. Furthermore, the proposed method yields improvements in sensitivity, specificity and reproducibility compared to existing methods. Finally, the proposed method is able to handle count (RNAseq) data from time course experiments as is shown on a real data set.

**Electronic supplementary material:**

The online version of this article (doi:10.1186/1471-2105-15-327) contains supplementary material, which is available to authorized users.

## Background

Cervical cancer is caused by infection with high-risk types of the human papillomavirus (HPV) followed by additional changes in the host cell genome. Insight in genes that are consistently altered over time will improve our understanding of the molecular mechanisms driving cervical carcinogenesis. These genes may provide novel biomarkers for early detection of cervical cancer as well as potential therapeutic targets. High-throughput techniques, such as microarrays and next generation sequencing, are tools for fast high-resolution genome-wide molecular profiling. Applying these techniques to measure genes at consecutive moments in time at multiple molecular levels generates a description of the occurrence of molecular abnormalities during cervical carcinogenesis.

A longitudinal *in vitro* system of four independent cell lines immortalized with either HPV16 or HPV18, previously shown to faithfully mimic cervical carcinogenesis at the (epi)genetic level [1–3], was used in this study. Cell lines were assayed for gene expression (mRNA) and gene copy number (DNA) with microarrays at consecutive moments in time, representing distinct stages of transformation. Abnormalities in DNA copy number were previously shown to directly affect expression of the genes located within these abnormalities and are believed to facilitate the identification of functionally relevant gene expression changes [4]. Integrating these two molecular levels will yield models of cancer development and progression, thereby reducing the complexity of (cervical) carcinogenesis [5]. We present a method that, in contrast to existing methods, is able to integrate DNA copy number and gene expression over time, while identifying temporal differential gene expression.

Available methods in current literature for time-course differential gene expression analysis can only be applied to a single molecular level. Since microarrays have become widely used for studying genome-wide gene expression, a range of statistical methods have been tailored for the identification of differentially expressed genes in microarray time-course experiments. Several of these methods are developed in an empirical Bayes framework [6–9]. Tai and Speed [9] use multivariate empirical Bayes statistics to rank time-course gene expression profiles. Their method is applicable to both single-condition and multiple-condition datasets and includes a variance stabilization imposing common matrix as a gene-specific variance-covariance matrix. Alternative approaches involve spline-based methods which fit a smoothed curve to the longitudinal data to use for statistical testing [10–12]. Storey et al. [11] use a population average time curve based on natural cubic splines to capture dynamics in gene expression levels and employ the F-test to identify significant genes. On the other hand, BATS [13] combines these two: it employs gene-wise functional modelling to explain temporal differential gene expression, which is casked in a hierarchical Bayesian framework.

In this article we present a method for identification of temporal differential gene expression driven by genomic abnormalities, introducing several new concepts. First, employing low-rank thin-plate splines and empirical Bayes shrinkage, identification of temporal differential gene expression is improved in terms of sensitivity, specificity and reproducibility. Second, including DNA copy number as a time-varying molecular covariate reduces residual variance and allows for the identification of genes which have variation in expression over time caused by genomic abnormalities. Genes with expression levels affected by DNA copy number aberrations have the capability to contribute to malignant cell growth [4, 14]. Identification of these genes is therefore essential for a better understanding of cancer development in general. Third, we impose a multivariate spatial prior for the DNA copy number effect to make the estimate more stable, borrowing information from neighboring features. Furthermore, by changing the link function our method can straightforwardly deal both with continuous and count data. To illustrate the wide applicability of our method we applied it to HPV-induced transformation (microarray) and head & neck cancer (RNA-seq) data.

## Methods

A method for the identification of temporal variation in gene expression (due to genomic abnormalities) from an integrative genomics study with a time-course set-up is presented. The variation in gene expression over time is described by a generalized linear mixed model employing low-rank thin-plate splines. Hyperparameters of the model are estimated from the data with an empirical Bayes procedure. With parameter estimates at hand, we describe how relevant hypotheses may be tested. The section concludes with extensions of the model and practical considerations for its application.

### Model

Consider a time-course microarray experiment where *n* cell lines are assayed repeatedly over  consecutive time points. At each time point both the DNA copy number and mRNA gene expression of each cell line are measured. Let the random variables *X*_*i*,*j*,*t*_ and *Y*_*i*,*j*,*t*_ represent the DNA copy number and the expression level, respectively, of gene *j* of cell line *i* at time point *t*. Their realizations are denoted by *x*_*i*,*j*,*t*_ and *y*_*i*,*j*,*t*_. For both random variables the index *j* runs from *j*=1 to *p*. This is due to the application of a matching procedure [15], which matches probes from two high-throughput platforms on the basis of their genomic location.

The expression levels of gene *j* are assumed to be normally distributed: , with  the error variance of the expression levels of gene *j*. The mean of this distribution is modelled as:
1

where *f*(·;·) and *h*(·;·) model the fixed and random effects, respectively, of the expression level of gene *j*. Both *f*(·;·) and *h*(·;·) are specified next.

The fixed effects, encompassing both cell line and DNA copy number effects, are modelled by a linear regression component:


where ***α***_*i*,*j*_ is the effect of cell line *i* in gene *j* and *β*_*j*_ the DNA copy number effect on the expression levels of gene *j*.

The random effect captures the dynamics in the expression levels over time and is modelled nonparametrically by low-rank thin-plate splines [16]. These splines provide enough flexibility for modelling gene expression variation over time. Furthermore, the low rank approximation avoids heavy computational costs due to a large number of unknown parameters. The random effect is then modelled by:


where **Z**_*t*_=(|*t*−*κ*_1_|^3^,…,|*t*−*κ*_*K*_|^3^) and ***γ***_*j*_=(*γ*_*j*_,…,*γ*_*j*,*K*_)^T^, the vector of coefficients of the spline. These coefficients are randomly distributed as . The *κ*_*k*_, *κ*_1_<*κ*_2_< ⋯<*κ*_*K*_, are fixed knots, equally distributed over the interval .

Model (1) is recasked as a semi-parametric mixed model. In a mixed model the random effects *γ*_*j*,*k*_ are assumed independent and all stemming from the same distribution . Within the context of thin plate splines this is achieved by assuming a particular parameterization of ***Σ***_*γ*,*j*_. This requires the definition of the matrix ***Ω*** with  for *k*_1_,*k*_2_=1,…,*K*. Furthermore, let  be the singular value decomposition of ***Ω*** with **U**_*ω*_ and **V**_*ω*_ containing its left and right singular vectors as columns and diagonal matrix **D**_*ω*_ of its singular values. It is then assumed that ***Σ***_*γ*,*j*_ can be written as . Under this assumption Model (1) can be reformulated as the mixed model:
2

where  and . The independent random variables *γ*_*j*,*k*_ and *ε*_*i*,*j*,*t*_ of this model are both multivariately normal with mean zero and covariances  only if *i*_1_=*i*_2_ and *t*_1_=*t*_2_ and zero otherwise, and . The choice of ***Ω*** results in a covariance matrix with higher covariances of random effects of neighboring knots (than those of more distant knots).

If we denote with **Y**_∗,*j*,∗_ matrix of measurements for gene *j* which are first ordered by cell lines *i* and within a cell line by time. The likelihood for gene *j* thus is:


which is to be used in the estimation.

### Estimation

The parameters of Model (2) are estimated by means of an empirical Bayes procedure. Empirical Bayes enables us to exploit the high-dimensionality of the data by ‘borrowing information across genes’, which yields more reproducible results. Information will be shared among genes via common hyperparameters of the priors of the model parameters. Here this sharing is done only for parameters that will be subject to inference. Other parameters are considered confounders that need to be taken into account but are not of central interest. In principle, our estimation allows common hyperparameters for these confounders, but at a computational cost.

For the fixed DNA copy number effect a Dirac-Gaussian mixture prior (a mixture of a point mass on zero and Gaussian distributions) is used:


where  denotes the Gaussian density with parameters (0,*τ*^2^). The point mass accommodates the proportion of genes without a DNA copy number effect. As this proportion is likely to comprise the majority of genes, it shrinks the *β*_*j*_ to zero for those genes with a gene dosage effect.

The random effect *γ*_*j*,*k*_ and error *ε*_*i*,*j*,*t*_ are endowed with normal priors:  and . Shrinkage is applied on the dispersion parameters  and  for two reasons: *a)* more stable parameter estimates and *b)* protection against over-fitting. The reciprocals of these parameters,  and , represent precision, for each a Gamma distribution is used as a conjugate prior. The parameters *a* and *b* of these hyperprior Gamma distributions are estimated using the method of [17]. The amount of shrinkage via this prior is determined by the data. The procedure is initiated with *a* and *b* resulting in a very flat prior on the precision. This corresponds to a very narrow prior on the variance of the random effect, that is, a flat spline. Iteratively, should the data give rise to it, the prior of precision becomes more informative. As a result the variance moves away from zero, increasing the flexibility of the spline. Would one desire more shrinkage our procedure allows the employment of a hyperprior composed of a Gamma and a point mass at zero.

For the fixed cell line effect *α*_*i*,*j*_ a Gaussian prior is assumed: . No shrinkage (via information borrowing) is applied to *α*_*i*,*j*_ as it is considered a confounder, for which an unbiased estimate is preferred.

We temporarily assume that the cell lines are merely biological replicates and no inferential statement with respect to the cell line effect will be made in the Section ‘Head-and-neck cancer’. Hence, for the moment the prior of the cell line effect *α*_*i*,*j*_ is . The variance of this prior depends on index *j*: the hyperparameter of this prior is different for each gene.

Given the hyperparameters shared by all genes, the model parameters of the individual genes are estimated by the mean of their posterior distributions. These are obtained by means of integrated nested Laplace approximations (INLA) [18]. INLA yields the marginal posterior distribution of a model parameter through integration of the posterior distribution over all remaining model parameters. This can be done computationally efficient by means of Laplace approximations under the assumption of a Gaussian prior on the model parameters. The use of approximated posterior distributions (instead of the possibly more exact posterior produced by Markov chain Monte Carlo (MCMC)) is motivated computationally: the posterior distributions of model parameters of many genes need evaluation.

It remains to choose the hyperparameters. An informed choice of the hyperparameters is made through application of the empirical Bayes procedure of [17]. That is, the hyperparameters are estimated from the data rather than set prior to the analysis. Apart from a less subjective choice of the hyperprior, this has two favorable consequences. First, it yields more reproducible results (confer the Section ‘Comparison’). Second, the hyperpriors employed cause shrinkage of the model parameter estimates. In particular, for the random effect it shrinks the dispersion parameter, which controls the smoothness of the fit [19]. Hence, it constrains the flexibility of the spline and thus reduces the risk of overfitting. An illustration of this effect on the fitted spline can be found in Additional file 1, Section 1.

The conventional empirical Bayesian estimate maximizes the product of the marginal likelihoods:
3

where *π*(·) denotes the prior of its argument (hyperparameters of the priors are suppressed for ease of notation). The hyperparameters of the priors of the ***α***_*j*_, *β*_*j*_, and  are estimated by maximization of loss function (3). Hereto equate the derivative of (3) with respect to the hyperparameters to zero and solve. The iterative procedure of [17] yields an approximate solution to estimating equation (3). The procedure of [17] is computationally fast and allows non-parametric hyperpriors.

From expression (3) it becomes clear how the parameter estimates are shrunken. The prior of (say) *β*_*j*_ is shared by all genes. Hence, the choice of the hyperparameters affects the posterior distribution of all *β*_*j*_. In particular, if the mass of *π*(*β*_*j*_) is more concentrated around zero, the posterior has more probability mass close to zero. Only if the data contains enough evidence (in favor of a non-zero *β*_*j*_) to outweigh the prior, the posterior will center around a non-zero value. The prior of *β*_*j*_ puts (via the spike at zero) more mass at zero. Moreover, the precision of the other mixture component is estimated from all genes. Under the assumption that a (vast) majority of genes do not exhibit an effect, the precision is under-estimated for the minority. This, together with the spike, yields a conservative prior leading to shrunken estimates.

Finally, we point out that the procedure described in [17] assumes covariates to be identical over features. In our setting the DNA copy number covariate varies over the features. It would be more appropriate to assume a different Dirac-Gaussian prior for each group of features that shares the same aberration pattern over the samples. These mixture priors differ in the variance of their Gaussian part. This could – in principle – be accommodated by a mixture of a point mass at zero and a lot of Gaussians, all with mean zero but different variances. However, a mixture of Gaussians with the same location but different variance may be approximated by a *t*-distribution with degrees of freedom equal to the number of Gaussians [20]. However, this approximation improves as the number of Gaussians in the mixture increases. On the other hand, a *t*-distribution with many degrees approaches a Gaussian. As there are usually many different genomic aberration patterns, we exploit this approximation and assume a common variance in our Dirac-Gaussian mixture prior. In a simulation study (Additional file 1, Section 2) we show that the *t*-distribution approximation does not differ substantially from the mixture of Gaussians with common mean but different variances.

### Hypothesis testing

From a biological point two questions are of main interest: *i)* does DNA copy number drive gene expression, and *ii)* is there differential expression over time? The former question can be answered by testing whether the DNA copy number effect *β*_*j*_ differs significantly from zero, evaluating the null hypothesis *H*_0_:*β*_*j*_=0 versus its alternative *H*_0_:*β*_*j*_≠0. The second question is addressed by testing  against the alternative . The alternative implies that there is at least one *γ*_*j*,*k*_≠0, resulting in a non-constant spline. Additionally, one may ask whether there is a difference between the cell lines, but this question is not considered here (although it could straightforwardly be addressed within the presented framework).

Both hypotheses are evaluated by means of the likelihood ratio statistic. For the first question on DNA copy number the statistic is:


where e.g.  is the estimate of ***α***_*j*_ under the alternative hypothesis *H*_*A*_. P-values are then obtained from the asymptotic (chi-square) distribution of the likelihood ratio statistics. The degrees of freedom of this chi-square distribution are equal to the difference in the number of parameters of the models compared. Note that this test is likely to be somewhat conservative, because  shrinks towards the null domain. The Dirac-Gaussian mixture prior, however, prevents overly conservative behavior, because it better separates the null genes from the non-null ones than a simple Gaussian prior. When testing , the degrees of freedom consumed by the penalized splines are estimated by the trace of hat matrix (details: Additional file 1, Section 3). Finally, to account for multiplicity the False Discovery Rate (FDR) is controlled by means of the procedure of [21].

This hybrid testing procedure (empirical Bayesian estimation with p-value based inference) mimics Limma [22, 23]. Limma obtains a shrunken (empirical Bayes) estimate of the variance and detects differential expression using a classical t-test (after adjusting the degrees of freedom). Here too we borrow strength (information) across genes to arrive at shrunken parameter estimates, and provide classical p-values. The latter meets the wishes of medical researchers. They commonly report p-values rather than Bayes factors. In addition, multiple testing corrections are generally more rigorous in a classical setting than in a Bayesian one, because the latter typically provides FDR estimation rather than control.

### Spatial multivariate prior

DNA copy number aberrations are often not confined to a single gene but span a large region of the genome that harbors multiple genes. Consequently, neighboring genes may share the same genomic aberration signature. At the transcriptomic level this results in co-expression of these genes [24]. Put differently, the DNA copy number effect (*β*_*j*_) of gene *j* may be correlated with that of neighboring genes (e.g. *β*_*j*−1_ and *β*_*j*+1_). This phenomenon is not accommodated by the aforementioned prior of the *β*_*j*_.

In this section we describe an extension of our procedure that incorporates the possible spatial correlation among the DNA copy number effects. To this end it is assumed that the *β*_*j*_ follows a first-order autoregressive (AR(1)) process along the genome: *β*_*j*_=*ρβ*_*j*−1_+*ε*_*j*_. The relevant parameter of this process is simply estimated by regression of the *β*_*j*_ on the *β*_*j*−1_.

Having obtained an estimate of the spatial correlation among the *β*_*j*_, it rests to refit the model. However, the assumption of an AR(1) process on the gene dosage effect complicates the refitting as this should now be done simultaneously for all *p* genes. This is computationally too demanding. To approximate the joint fit the model is refitted per triplet of neighboring genes (e.g. for *β*_*j*−1_,*β*_*j*_,*β*_*j*+1_). For each triplet a trivariate normal prior is assumed (all other priors as before):


where the correlation structure of the covariance matrix follows an AR(1) process. For the re-estimated vector of *β*_*j*_s only the middle one is conserved. More details are provided in Additional file 1, Section 4.

Besides doing more justice to the underlying biology the ‘spatial prior’ above reduces the variation of the DNA copy number effect. This is achieved as the assumption of an AR(1) process effectively ‘averages’ the DNA copy number effect over neighbouring genes. As such, it is also a way of borrowing information across genes.

### Practical considerations

**Figure 1:**
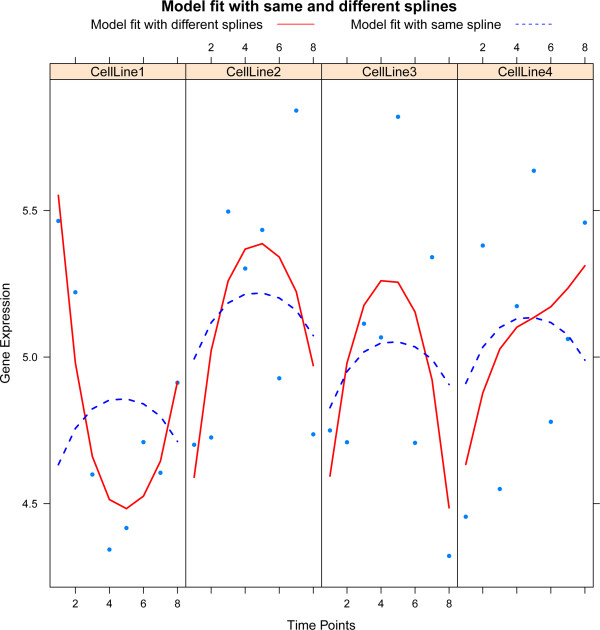
**Illustration of the same and different spline model.** Each panel, one per cell line, plots gene expression against time. The solid red curve represents the fit of the model with a different spline per cell line, while the dashed blue lines depict the fit of the same spline model.

A straightforward extension of Model (2) is to allow for a different spline in each cell line. This reflects the biological plausibility of different dynamical behaviour in different cell lines. In particular, we may then test for differences in the behavior over time between the cell lines. When rewriting Model (2) to a vector notation, the incorporation of different splines per cell line amounts to the replacement of  by  (the operator ⊗ is the Kronecker product), and adjusting the parameter vector ***γ***_*j*_ accordingly. Figure 1 illustrates the difference between the same and different spline models.

Within mixed Model (2) DNA copy number and time (via the spline) compete to explain the gene expression. Due to its flexibility the spline may consume variation in expression levels actually due to DNA copy number changes. Moreover, with DNA copy number changes being a more clearly delineated cause (than time in the form of a nonparametric spline), we prefer to attribute variation in expression levels to the genomic aberration. To let DNA copy number changes prevail over the spline, the design matrix **Z** of the latter is orthogonalized to the DNA copy number data. This orthogonalization does not affect the overall fit, but ensures that the spline captures only variation in expression levels that cannot be explained by DNA copy number changes. The effect of the orthogonalization is illustrated in the Section ‘HPV-induced transformation’.

To determine the optimal number of knots, we employ the deviation information criterion (DIC) [25]. The DIC is a measure for the balance between fit and complexity. For each gene the DIC of the model is estimated for different numbers of knots. The number of knots that over all genes yields the best DIC is chosen as the optimal *k* and applied to all genes in the analysis. More details are provided in Additional file 1, Section 5.

## Results and discussion

### HPV-induced transformation

The proposed method is demonstrated on data of an experiment on HPV-induced transformation. The experiment intends to faithfully mimic cervical cancer development employing a HPV-immortalized *in vitro* cell line model. Hereto two cell lines are affected with HPV16 and two with HPV18 [26]. Over time these cell lines acquire genomic and transcriptomic changes. To assess these changes, the genomic and transcriptomic characteristics of the cell lines are measured at eight time points by means of oligonucleotide microarrays. The preprocessing of the DNA copy number data comprises of median normalization and segmentation using the circular binary segmentation (CBS) method [27]. Similarly, the gene expression data are background corrected using robust multi-array average (RMA) [28] and between-array normalized by the robust quantile method. Finally, the resulting expression intensity values are transformed using the variance stabilizing transformation [29]. DNA copy number is assigned to each transcript from the expression array by the overlapPlus matching procedure of [15] which uses chromosomal location information. The final data set contains genomic and transcriptomic information on 37768 features, however in this section analysis is performed only on one chromosome which contains 2202 features.

We now turn to the identification of genes with differential expression over time. To this end only the expression data features are used, ignoring the effect of genomic aberrations in Model (2). Gene *j* exhibits temporal gene expression if  is rejected. This corresponds to a spline differing from a flat line, and thus indicating changes in expression levels over time. Temporal gene expression is identified both with a common and different spline(s) for the cell lines. In both analyses the optimal number of knots equals two (determined by the procedure described in the Section ‘Practical considerations’). Specification of the prior distribution and description of the hyperparameter estimations for  can be found in the Section ‘Estimation’.

Table 1 (the first row) shows the number of features that exhibit significant temporal differential expression at a 5% false-discovery rate. Considerably more significant genes are identified when allowing for a different spline per cell line. This is in line with expectations as it is the more flexible model. Often it also does more justice to the biology, as many of its selected genes indeed show different behavior over time among cell lines. The common spline model may yield a poor fit for these genes, in particular if there is opposing behavior among the cell lines. However, the common spline model is effective in identifying genes which are consistently up- or down-regulated in all cell lines. The model with a common spline found 74 significant features not identified by the model with a different spline per cell line. In the latter analysis these genes have *p*-values close to but just short of the significance threshold.

**Table 1:** **The number of significant probes identified in the analysis for temporal differential expression and copy number (CN) effect**

		Same spline		Different spline	
Effect	Model	Standard	Orthogonal	Standard	Orthogonal
Time	Splines	417		583	
	CN+Splines	204	203	421	421
CN	CN+Splines	402	403	380	380
	Multivariate	398	399	377	380

Analysis is performed only on 2202 features, which represent one chromosome.

The common and different spline models employed for the identification of temporal differential expression are now extended to include DNA copy number (as originally proposed in the Section ‘Model’). As noted in the Section ‘Practical considerations’ the flexibility of the spline may consume part of the DNA copy number effect. The proposed remedy limits (via projection) the spline basis to the space orthogonal to space spanned by the DNA copy number information. To assess the potential gain of the orthogonalization each analysis, with common and different spline(s), is done with and without orthogonalization. Prior distributions are as before. The number of knots is determined as done previously (optimal number still equals two). For each analysis hyperparameters of DNA copy number and spline(s) are re-estimated by the empirical Bayes procedure.

We first discuss the number of features with differential temporal expression (given in the second row of Table 1). As in the analysis without DNA copy number, the different spline model identifies substantially more features than the model with a common spline for the cell lines. Orthogonalization of the (common) spline basis onto the DNA copy number data misses one feature in comparison to the non-orthogonalized analysis. This feature is found only with the non-orthogonalized spline basis. In the latter analysis it only passes the significance threshold by a small margin. Hence, in these data the effect of orthogonalization on the identification of temporal differential expression is limited.

**Figure 2:**
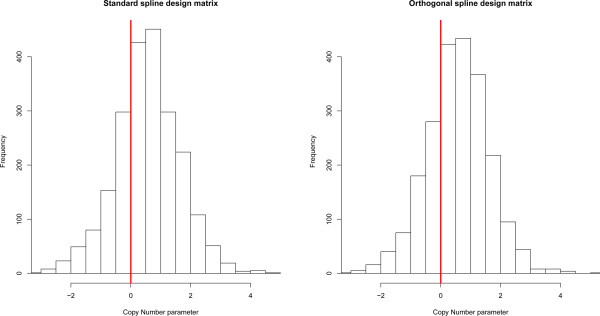
**Histograms of DNA copy number parameters.** The left panel represents DNA copy number parameters estimated using standard spline design matrix, while the right panel indicates parameters estimated employing an orthogonalized spline design matrix.

Turning to the effect of DNA copy number, we first analyzed the data with Model (2) containing only the fixed cell line and DNA copy number effect. This analysis identified 568 features with a significant gene dosage effect on expression. Inclusion of the time effect in Model (2) reduces the number of features with a significant gene dosage effect on expression (third row of Table 1). This is due to the fact that the spline competes with DNA copy number to explain the variation in expression levels: the former (being more flexible) captures variation caused by the latter. That aside, here too we see that the improved fit (now due to the increased flexibility of a different spline per cell line) yields a surge in the number of findings. The orthogonalization of the spline basis identify only one additional feature (using common splines) compared to the standard analysis. The effect of orthogonalization is more visible in the gene dosage effect *β*_*j*_, as can be witnessed from Figure 2. Clearly, orthogonalization moves the distribution of the *β*_*j*_’s to the right (the positive domain, which corroborates with the biologically expected direction of the effect). Finally, on the full data set (not shown) the analysis using the orthogonalized spline basis gives a modest improvement in the number of genes significantly affected by DNA copy number.

**Figure 3:**
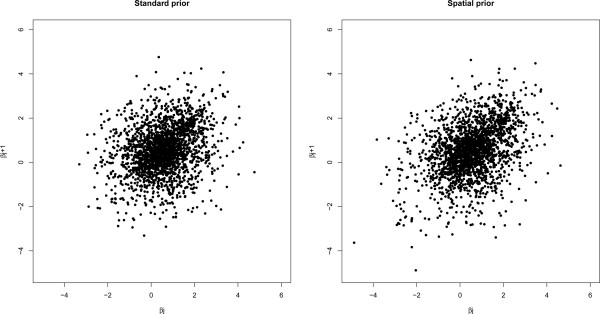
**Partial correlation of DNA copy number parameter, where** ***β***_***j***_ **is plotted against** ***β***_***j*+1**_ **.** In the left panel parameters of DNA copy number *β*_*j*_ are estimated univariate, while in the right panel parameters are estimated multivariate imposing the spatial prior.

**Figure 4:**
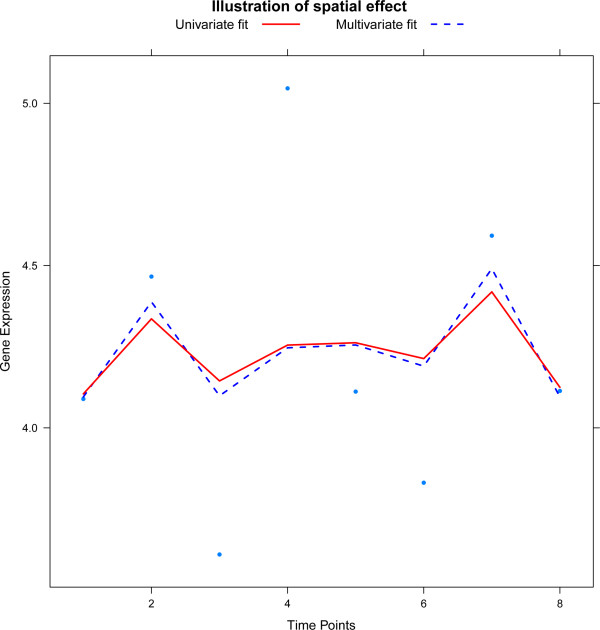
**The plot illustrates the effect of the spatial prior for one gene in a single cell line.** Gene expression is plotted against time and lines represent the univariate (red, solid line) and multivariate fit (blue, dashed line).

The effect of DNA copy number changes is also analyzed with the spatial prior discussed in the Section ‘Spatial multivariate prior’. This prior aims to capture roughly the spatial dependency between consecutive features, thus hoping to do more justice to the underlying biology. Clearly, estimation of the gene dosage effects with the spatial prior improves the lag one partial correlation among these effects (confer Figure 3). Changes in the actual parameters are noticeable but small. This has limited effect on the fit of the model. Consequently, the significance analysis of the DNA copy number effect identifies almost the same number of significant features (fourth row of Table 1). Spatial prior imposed reduces the variation among contiguous features as, effectively, it has a smoothing effect on estimated DNA copy number effects. Figure 4 illustrates the spatial effect by showing the fit of the model with and without spatial priors on the DNA copy number parameter (in Additional file 1, Section 7 illustrates this effect in all four cell lines). As in the temporal differential expression analysis, the number of features identified with standard or orthogonal spline basis hardly differs.

Another striking feature of Table 1 is the difference between the number of significant features with differential temporal expression and those with a gene dosage effect: the former exceeds the latter (in case of the full model with different splines per cell line). In part this difference is explained by the flexibility of the splines. But also by the presence of many other regulators of gene expression (e.g. microRNA, methylation, transcription factors) that result in temporal differential expression are captured by the splines. On the other hand, comparing the analyses with a common spline more significant gene dosage effect than temporal differential expression is found. This is due to the fact that DNA copy numbers may strongly correlate with gene expression over time. Features with expression levels that do not consistently (over cell lines) co-vary with DNA copy number are missed by the common spline model (and not including the gene dosage effect).

**Figure 5:**
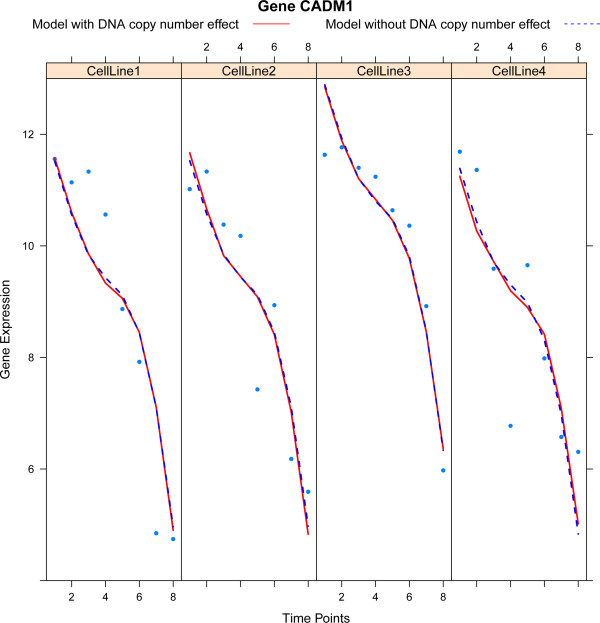
**Expression levels of CADM1 over time.** The solid red and dashed blue lines are the fits of the model with and without DNA copy number parameter, respectively.

To give some more tangible insight into the results from the analyses above, we single out the CADM1 gene. It is among the genes with the highest significance for temporal differential expression (using Model (2) with a common spline). Indeed, CADM1 is down-regulated in all four cell lines over time (confer Figure 5). This gene is well-known in cervical cancer to be down-regulated during progression due to an increasingly methylated promoter region [1, 30]. The fits of Model (2) with and without DNA copy number hardly differ. The estimate of the DNA copy number parameter (obtained with the common spline model) confirms this (). This suggests that the down-regulation of CADM1 is not due to a DNA copy number loss, which corresponds with previous findings (e.g. [1]).

**Figure 6:**
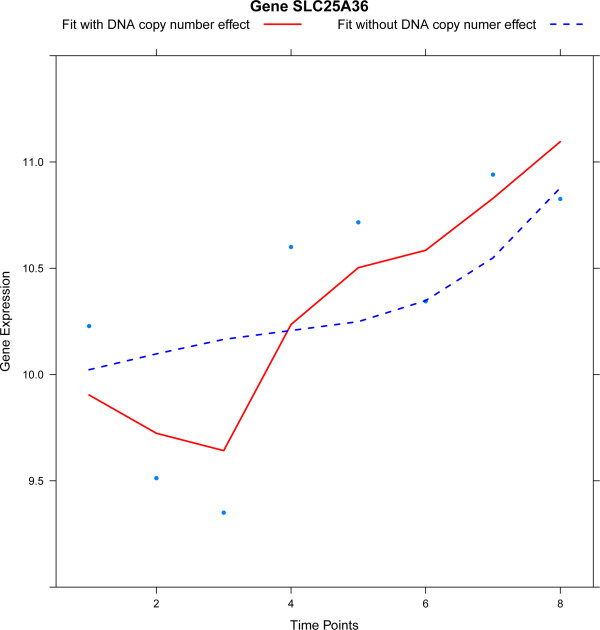
**Expression levels of SLC25A36 over time in a single cell line.** The solid red and dashed blue lines are the fits of the model with and without DNA copy number parameter, respectively.

To contrast the results for CADM1, we focus on the SLC25A36 gene. SLC25A36 exhibits temporal differential expression (irrespective of the choice for common or different spline model). SLC25A36 is also identified as a gene with a significant DNA copy number effect with estimate . Inclusion of DNA copy number in the model improves the fit substantially (confer Figure 6, illustration on all four cell lines in Additional file 1, Section 7). This is seen in Figure 6 as the difference in fit for the model with and without DNA copy number. These results for SLC25A36 corroborate with existing medical literature: the gene dosage effect for this gene has already been reported in cervical cancer [31].

**Figure 7:**
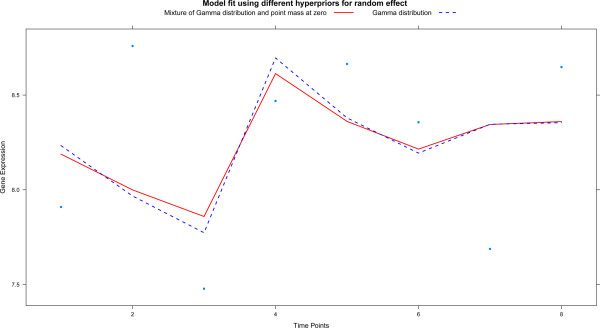
**This plot illustrates the effect of using different priors in one cell line.** Gene expression is plotted against time (single cell line only). The solid red line is the fit of the model with a standard prior, while the dashed blue line is that of the model with an alternative prior.

Finally, we want to assess the sensitivity of the results with respect to the choice of the prior distribution. For illustration purposes we focus on the hyperprior of the random effect *γ*_*k*,*j*_. In the Section ‘Estimation’ we suggest to use the Gamma distribution. However, we have also implemented a mixture of a point mass at zero and Gamma distribution, which one expects to lead to more shrinkage. Model (2) using different splines and a standard design matrix is refitted now with this mixture prior for the random spline effect. Application of our empirical Bayes procedure with the Gamma prior identified 421 features, while the Dirac-Gamma mixture prior selected 396 features. The latter 396 are all included in the former 421 features. The slight reduction in the number of selected features is of course due to the inclusion of the point mass at zero. The fit of both resulting models is almost identical for most features, but for some features with a slightly less flexible spline as in Figure 7 (Additional file 1, Section 7 illustrates effect in all four cell lines).

### Head-and-neck cancer

To illustrate the wide applicability of our framework, we present the analysis of sequencing data from a head-and-neck cancer study endowed with a time-course set-up. Oshlack et al. [32] noticed that there are currently no appropriate methods for the analysis of RNA-seq data from time-course experiments. The head-and-neck study aims to identify temporal differential expression due to overexpression of a particular microRNA. MicroRNAs are small 20-22nt non-coding RNAs that inhibit expression of their target genes. The microRNAs recognize these genes by their seed sequence that is complementary to a sequence in the 3’ untranslated region (UTR) of the target transcripts. One gene can be targeted by multiple microRNAs and one microRNA may target a multitude of genes. It is therefore not simple to find a specific relevant target gene of a microRNA. In a previous functional screen microRNAs were identified that specifically kill head and neck cancer cells, but not normal cells [33]. The respective target genes of these microRNAs were to be identified in a follow-up experiment. In this follow-up experiment cells of a squamous cell carcinoma cell line were transfected by a microRNA mimic and a control. The transfected cells were grown *in vitro* and sampled at six time points. Transcript levels of the 2×6 samples were sequenced. Data were mapped to the human genome and raw count data (reads) per gene transcript were used and not summarized per gene. Their normalization comprises rescaling by a the trimmed mean of each sample’s library (following [32]). Normalized data are rounded to the closest integer to retain the count interpretation of the data.

Model (2) cannot be directly applied to the sequencing data, as the normal distribution is often a poor approximation for the distribution of counts. The normality assumption is replaced by the (zero-inflated) negative binomial [17, 34]: -. The mean *μ*_*i*,*j*,*t*_ of the counts is (after transformation by the inverse of the link function) still modeled by the right-hand side of Model (2) with assumptions on model parameters in place. Hyperparameters are then estimated via the empirical Bayes procedure previously described.

**Figure 8:**
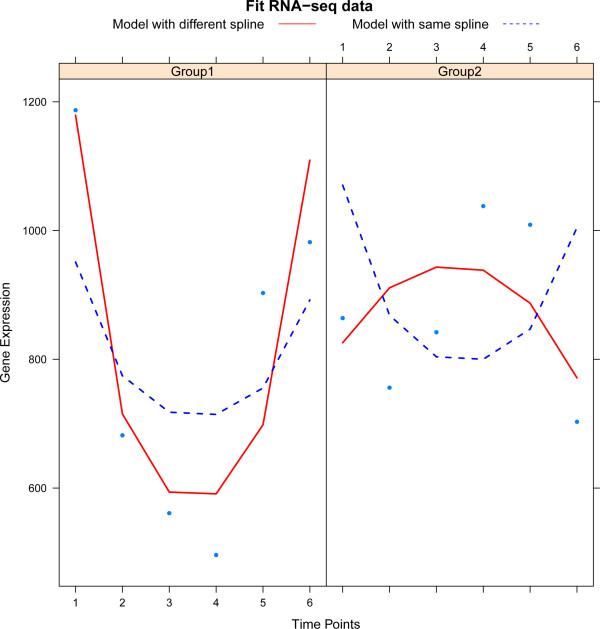
**The dots represent the RNA-seq tag counts plotted against time.** The solid (red) line represents the fit of the model with different splines per group while the dashed (blue) line that of the model with a common spline for both groups.

The analysis of the head-and-neck cancer data concentrates on two main questions: identification of tags with temporal variation and those different between the two conditions. To answer this, Model (2) is used without the DNA copy number term (which is not included in the experiment). Common and different spline models are employed as in the Section ‘HPV-induced transformation’. Parameter *γ*_*j*_ is the main parameter of interest and the analysis compares the model with and without time effect. The optimal number of knots (again two, for both models) is determined using the procedure described in the Section ‘Practical considerations’. Prior distributions for cell line and time effect are as in the Section ‘Estimation’. Hyperparameters are estimated for each analysis separately, but only the variance of the random time effect is shrunken via the empirical Bayes procedure. Counts of are fitted using the model with same and different splines as illustrated for one RNA-seq tag in Figure 8.

The number of tags with a significant (at the 5% FDR level) temporal variation identified equals 8416 (10951) for the common (different) spline model. As observed in the analysis of the HPV-induced transformation data, the use of a different spline leads to many more findings. Again, this is explained by the improved fit due to a more flexible model. In particular, all the tags identified with the same spline model are also found by its flexible counterpart.

### Comparison

The proposed method is compared to three well-known alternatives for significance analysis of time-course microarray data: EDGE, [11], timecourse, [9], BATS [13, 35], and a reference method. The reference method comprises a standard frequentist approach. It fits a linear mixed-effect model, while the null hypothesis is evaluated through an analysis of variance approach (anova), comparing two nested models (with and without random effects).

These competitors have not been designed for the analysis of integrative genomics studies with a time-course set-up. Hence, our method is applicable to a wider class of studies. Besides this qualitative argument, we wish to have a quantitative comparison of the methods. To this end the comparison is restricted to time-course genomics studies involving only a single molecular level. Moreover, to avoid bias of any of the methods by a particular model choice, the comparison is done on two real data sets. The first is the HPV-induced transformation data from the Section ‘HPV-induced transformation’, limited to the gene expression levels only. The other data set is included in the EGDE-package [11], where gene expression has been monitored in four individuals from a control and endotoxin-treated group. Samples have been collected at five different time points: 2, 4, 6, 9 and 24 hours after treatment. One individual lacks data for the control group at two time points (4 and 6 hours). Since the timecourse method cannot deal with missing time-points this individual is omitted from the analysis. At each time point expression levels of 800 genes are available.

We now briefly describe the other methods used in the comparison: EDGE, BATS and timecourse. For a more detailed description please refer to the corresponding references.

EDGE ([11]) captures the temporal variation in the expression levels of gene *j* by means of a p-dimensional B-spline basis. Temporal differential expression is evaluated by an F-statistic measuring the goodness-of-fit of the null hypothesis (a flat or constant spline) in comparison to the alternative hypothesis. In the comparison EDGE is used with default parameter settings.

Method timecourse ([9]) uses novel multivariate empirical Bayes statistic to rank time-course gene expression profiles. Gene expression in timecourse method is assumed to follow a multivariate normal distribution with gene-specific mean and covariance. Conjugate priors are assumed on the unknown parameters. Hyperparameters of the conjugates are estimated from the data. Timecourse yields stable variance estimates by borrowing (co)variance information across genes. The posterior distribution and test statistics are obtained in an analytic form. Genes may be ranked using either Hotelling *T*^2^ or *MB*-statistics. For the comparison we used the timecourse R-package with standard settings and Hotelling *T*^2^-statistic, due to the balancedness of the study design (equal number of replicates per gene).

Finally, BATS ([13]) which combines characteristics from previously described methods. Similar to EDGE gene expression variation over time is modelled by a polynomial function, while imposing a hierarchical Bayesian model on the parameters (as timecourse). BATS is flexible in its choice of the prior for dispersion related parameters, it offers delta, inverse Gamma and exponential priors. Significance analysis is based on the genes’ Bayes factors, while multiplicity correction is addressed in Bayesian manner ([36]).

Sensitivity and specificity of the four aforementioned methods are compared in both data sets. Hereto knowledge of the genes with true temporal differential expression is needed. In its absence we constructed a consensus set which fulfills this role. That consensus set comprises of the features identified by all four methods. Sensitivity is then the proportion of features with temporal differential expression correctly identified as such. On the other hand, specificity is the proportion of features which are correctly identified as features without differential expression over time (hence, rightly not significant). Sensitivity and specificity of each method are assessed for various numbers of significant features.

**Figure 9:**
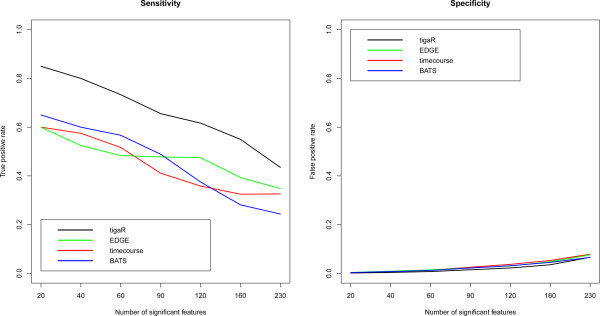
**Comparison of sensitivity and specificity for tigaR, EDGE, BATS and timecourse on the data set of the Section ‘HPV-induced transformation’.** The left (right) panel displays the sensitivity of the methods (specificity) by plotting true (false) positive rate against the number of significant features.

Figure 9 presents the resulting sensitivity and specificity for the HPV-induced transformation data. The left panel of Figure 9 compares the sensitivity. While BATS, timecourse and EDGE are more or less on a par, they all have a lower true positive rate than tigaR. With respect to the specificity, the methods are more or less on a par with tigaR having a slightly lower false positive rate than the other methods. This is confirmed (though much less pronounced) by the results from the EDGE-package data (see Additional file 1, Section 6). For both sensitivity and specificity the methods perform similarly with tigaR having a marginal lead.

Furthermore, we compared the reproducibility of the five methods (now including reference method). Hereto each data set was divided into two equally sized groups. We assessed how well the results of the two splits coincided. This boils down to the application of each method on both splits. The overlap in significant features for each method was determined.

**Figure 10:**
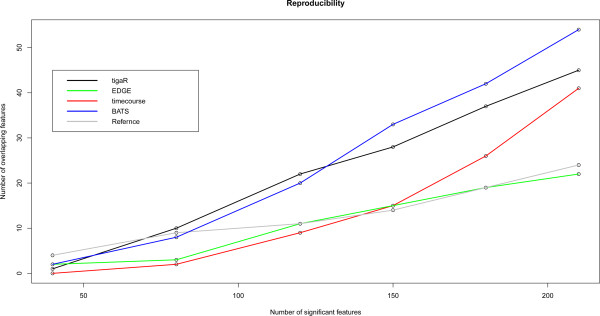
**Reproducibility of tigaR, EDGE, BATS, timecourse and reference model are assessed on the data set of the Section ‘HPV-induced transformation’.** The number of significant features identified in both groups are plotted against the initial number of features.

Figure 10 shows the reproducibility of each method on the data set from the Section ‘HPV-induced transformation’. It reveals that BATS and tigaR reproduce substantially better than the competitors. This is confirmed in the other data set (see Additional file 1, Section 6). The superior reproducibility is most likely a consequence of the empirical Bayes approach (borrowing of information stabilizes estimates) in tigaR and very informative priors in BATS. The tigaR, BATS and timecourse-methods do much better than EDGE in both data sets. The former three all exploit the Bayes principle (in different ways though) which improves estimates of the variance parameters, while EDGE does not.

## Conclusions

We presented a method for the analysis of integrative (onco) genomics studies with a time-course experimental design. The method identifies temporal differential gene expression while accounting for time-varying molecular covariates like DNA copy number changes. Simultaneously, the method assesses which of these covariates significantly contributes to temporal differential gene expression. The method employs a mixed model describing the temporal changes in gene expression in terms of DNA copy number and a (low-rank thin-plate) spline which captures additional temporal variation in the transcript levels. The method estimates the parameters of this model by means of an empirical Bayes procedure that ‘borrows information’ across genes. The empirical Bayesian procedure shrinks the parameter estimates (towards zero), thus accounting for multiplicity. This shrinkage enhances the reproducibility of the results. In a direct comparison with other methods for the identification of temporal differential expression, the proposed method proved to be a strong competitor, particularly in terms of reproducibility. In addition existing methods cannot incorporate additional genomics data. Furthermore, our method is straightforwardly applicable to count data resulting from RNA-seq experiments. Application to an integrative oncogenomics study, involving HPV-transformed cell lines, confirmed genes CADM1 and SLC25A36, known to be implicated in the development of cervical cancer. The presented methodology also identified other, novel and potentially interesting genes. These are currently under investigation and will be reported in a follow-up medical paper. Preliminary pathway analysis already showed that genes identified from this dataset by tigaR but not by the other methods were enriched for genes involved in cellular transformation.

Our ongoing research concentrates on two extensions of the proposed method. First, we are considering the inclusion of microRNA data. MicroRNAs affect expression levels post-transcriptionally. However, which microRNA targets which mRNA is only partially known. Hence, integration of temporal microRNA expression data also needs to address the problem of selecting the microRNAs targets. With the number of microRNAs known and typically measured in time-course integrative genomics studies being larger than the number of samples (# time points × # cell lines) this adds an additional layer of complexity to the problem.

The second extension comprises the integration of pathway information. This requires a multivariate formulation of the model for temporal changes in gene expression. Next to DNA copy number changes now the changes in transcript levels of other genes in the pathway may need to be included. A key challenge here is to ‘borrow information’ within and between pathways.

The methodology described in this paper is implemented in the R-package tigaR available upon request from the first author (v.miok@vumc.nl).

## Electronic supplementary material

Additional file 1: **Supplementary material.** Supplementary document containing details about the simulation study setup, additional figures and tables. (PDF 1013 KB)
