# Supplementary material for: tigaR: integrative significance analysis of temporal differential gene expression induced by genomic abnormalities
Source: BMC Bioinformatics. 2014 Oct 2;15(1):327. doi: 10.1186/1471-2105-15-327 (PMC4288633; doi:10.1186/1471-2105-15-327)
Supplement: Supplementary file 1 — Additional file 1: Supplementary material. Supplementary document containing details about the simulation study setup, additional figures and tables. (PDF 1013 KB) [file 12859_2014_6640_MOESM1_ESM.pdf]

SUPPLEMENTARY MATERIAL
tigaR: Integrative significance analysis of temporal differential gene expression induced by genomic abnormalities

Viktorian Miok[1,2], Saskia M Wilting[2], Mark A van de Wiel[1,4], Annelieke Jaspers[2], Paula I van Noort[5], Ruud H Brakenhoff[3], Peter JF Snijders[2], Renske DM Steenbergen[2], Wessel N van Wieringen[1,4]

[1]*Department of Epidemiology & Biostatistics,* [2]*Department of Pathology and* [3]*Department of Otolaryngology/Head-Neck Surgery, VU University Medical Center, P. O. Box* 7057, 1007 *MB Amsterdam, The Netherlands.*

[4]*Department of Mathematics, VU University Amsterdam, De Boelelaan* 1081*a,* 1081 *HV Amsterdam, The Netherlands.*

[5]*InteRNA Technologies BV, Padualaan 8, 3584 CH Utrecht, The Netherlands.*

1 Effect of shrinkage

The proposed empirical Bayes procedure shrinks the dispersion-related parameters to constrain flexibility of the spline(s) and protect against overfitting. Shrinkage on the random effects $\gamma_{k,j}$, which control the smoothness of the fit, is achieved via the choice of the hyperprior. Here we investigate the effect of this choice. Two different hyperpriors are considered: the Gamma distribution and a mixture distribution of the Gamma and a point mass at zero. The point mass at zero in the latter hyperprior is expected to yield more shrinkage. We compare the fit (of the splines) with both hyperpriors. Figure 1 illustrates the difference between the fits (using the two hyperpriors). The difference between the fits is not dramatic, but it shows (as expected) that the mixture of Gamma and point mass at zero yields splines with decreased smoothness.

The decreased smoothness as an effect of including point mass at zero, which enforce stronger penalization of non-zero random effects, is considered in the sensitivity and specificity analysis. Figure 2 shows the sensitivity and specificity compared among the methods tigaR, EDGE, timecourse, BATS and tigaR (now employing a ‘Gamma plus point mass at zero’ hyperprior). From Figure 2 we conclude that less smooth splines does not substantially affect the sensitivity and specificity of the method. Thus, the Gamma hyperprior guards (sufficiently) against over-smoothing and potential over-fitting.

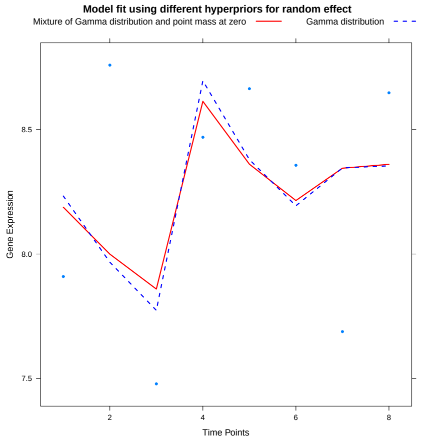

Figure 1: The effect of using different hyperpriors in a single cell line. Gene expression is plotted against time (single cell line only). The solid red line is the fit of the model with a standard hyperprior, while the dashed blue line is that of the model with the alternative mixture hyperprior.

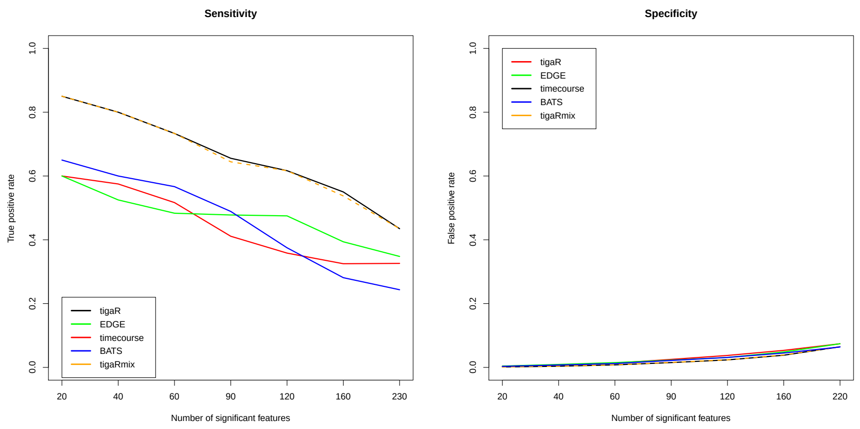

Figure 2: Plots represent comparison of packages tiagR, EDGE, BATS, timecourse and tigaR which employ hyperprior composed of Gamma and point mass at zero for the sensitivity and specificity on HPV-induced transformation data. The left panel represents the sensitivity (of each model), where the true positive rate is plotted against the number of significant genes. The right panel illustrates the specificity (of each method), where the false positive rate is plotted against the number of significant genes.

2 Effect of a gene specific covariate on shrinkage

DNA copy number data varies from one chromosome to another, but within a single chromosome these data exhibit genomic regions that share the same aberration patterns over the samples. We assume a common hyperprior for the gene dosage effect within and between such regions. This is justified within a region, but between regions it may be more appropriate to assume a different dirac-Gaussian hyperprior. By simulation we show that the effect of assuming a common hyperprior for all regions is not unreasonable, in the sense that it does not yield results that differ much from those obtained when employing a separate hyperprior for each region.

The simulation involves the data set described in Section 2.1. For 15 regions (comprising features with a common DNA copy number pattern over the samples) we estimate parameters of the gene dosage effect prior distribution with both separate Dirac-Gaussian hyperpriors and a common one, employing the procedure described in Section 1.2. First, we estimate the hyperparameters of the DNA copy number effect using a different Dirac-Gaussian hyperprior for each region. Then, we also estimate hyperparameters of the gene dosage effect using a dirac-Gaussian hyperprior common to all regions. Figure 3 shows the histograms of the sampling distribution from the averaged (over the regions) prior and the common prior of the gene dosage effect. At first glance the histograms are similar. A better comparison is provided by the Quantile-Quantile plot (right panel of Figure 3). The Q-Q plot reveals that these prior distributions (with separate and common Dirac-Gaussian hyperpriors) are alike around their modes, but differ somewhat in their tails. This difference in the tails will decrease as the number of regions increases. In most data sets the number of regions easily exceeds a hundred yielding a much better approximation

in the tails. On the other hand, a mixture of many Gaussians with common mean but different variances may be approximated by a t-distribution with the degrees of freedom equal to the number of Gaussians in the mixture. Furthermore, as the degrees of freedom of a t-distribution increase it approaches a Gaussian. Hence, a better approximation in the tails is expected with more regions.

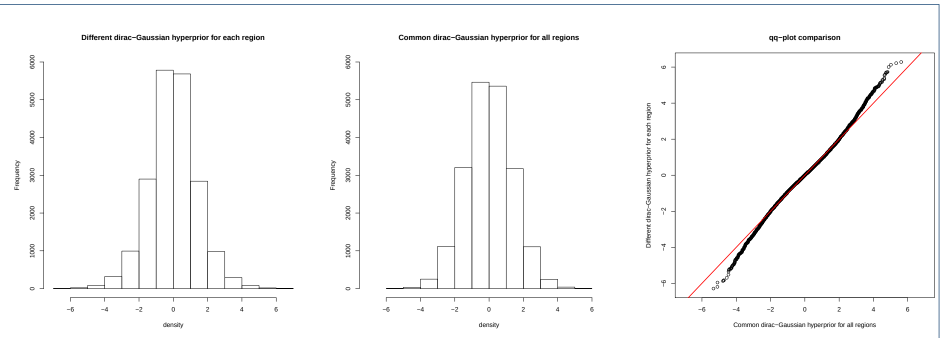

Figure 3: Comparison of common and separate Dirac-Gaussian hyperpriors. The left panel depicts the prior distribution of the gene dosage effect using a common Dirac-Gaussian hyperpriors for all regions. The middle panel represents the prior distribution for different Dirac-Gaussian hyperpriors per region. The right panel compares these distributions using a Q-Q plot.

3 Degree of freedom estimation for penalized splines

Within the context of generalized linear mixed models the effective degrees of freedom are (usually) defined by the trace of the hat matrix. The fit of the mixed Model (2) per gene j is obtained by $\hat{\mathbf{Y}}_j = \mathbf{H}_{\lambda,j}\mathbf{Y}_j$, where $\mathbf{H}_{\lambda,j}$ is the hat matrix, which projects the data onto the space spanned by the design matrix. The matrix $\mathbf{Y}_{*,j,*}$ is transformed into a vector by the vec-operator: $\mathbf{Y}_j = \text{vec}(\mathbf{Y}_{*,j,*})$, where the measurements of gene j are first ordered by cell lines i and within a cell line by time. The hat matrix for Model (2) is defined using smoothing parameter $\lambda = \sigma^2_{\gamma,j}/\sigma^2_{\varepsilon,j}$, which controls the smoothness of the fit. Let $\mathbf{C}_j$ be the joint design matrix comprising covariate information for both the fixed and random effects. The hat matrix is then:

$$\mathbf{H}_{\lambda,j} = \mathbf{C}_j \left(\mathbf{C}_j^{\mathrm{T}}\mathbf{C}_j + \lambda\mathbf{I}\right)^{-1} \mathbf{C}_j^{\mathrm{T}}.$$

We exemplify the use of the matrix $\mathbf{C}_j$. Consider the data set from Section 2.1 using a model with a common spline. Then, $\mathbf{C}_j = [\mathbf{W}_{i,t}|\tilde{\mathbf{Z}}_t]$, where $\mathbf{W}_{i,t}$ is a matrix with cell line and DNA copy number covariate information, while $\tilde{\mathbf{Z}}_t$ contains that for the random (time) effect. Matrix C_j has dimensions 32×7, where 32 corresponds to the number of observations on gene j, while 7 represents the number of covariates: four cell line effects, one gene dosage effect, and two for the spline (a common spline with two knots). The rows of $\mathbf{C}_j$ are ordered as those of $\mathbf{Y}_j$.

Generalizing the definition of degrees of freedom (as done in [1]), it is defined as the trace of the hat matrix: $\text{df} = \text{tr}(\mathbf{H}_{\lambda,j})$. This is thus the degree of freedom of mixed Model (2), depending on the smoothing parameter λ. It can be interpreted as the number of fitted parameters. Thus, df indicates the 'amount of fitting' done by $\mathbf{H}_{\lambda,j}$, which is strictly monotone in λ.

4 Parameter estimation using the spatial prior

In this section we present how parameters of Model (2) are re-estimated when imposing a spatial prior on the gene dosage effect. First, all hyperparameters of Model (2) are estimated univariate as described in Section 1.2. Then, hyperparameter ρ (of the spatial prior) is estimated by regressing β_j on β_{j-1} (justified by the assumption that β_j follows a first-order autoregressive process). Combining the univariate estimated hyperparameters $\sigma^2_{j-1}, \sigma^2_j, \sigma^2_{j+1}$ with the estimated spatial hyperprior parameter ρ, yields the covariance matrix of the spatial hyperprior of a contiguous triplet. With this estimated spatial prior and the other (univariate) priors at hand, model parameters are re-estimated trivariately (per triplet of contiguous genes). Due to the fact that INLA does not allow to estimate a trivariate model with an AR(1) covariance structure on a fixed effect, estimators of model parameters are derived analytically.

Analytical estimators are derived separately for fixed and random effects. For convenience of notation we write $\mathbf{W}_{i,j}$, the joint design matrix of the cell line and DNA copy number information, and $\boldsymbol{\theta}_j = (\boldsymbol{\alpha}_{i,j}, \beta_j)$, the vector of corresponding parameters. Further, the prior distributions for fixed and random effects can be written as $\boldsymbol{\theta}_j | \sigma^2_{\theta,j} \sim \mathcal{N}(\mathbf{0}, \sigma^2_{\theta,j}\boldsymbol{\Sigma})$ and$\boldsymbol{\gamma}_j | \sigma^2_{\gamma,j} \sim \mathcal{N}(\mathbf{0}, \sigma^2_{\gamma,j}\boldsymbol{\Psi})$, for appropriate covariance matrices $\boldsymbol{\Sigma}$ and $\boldsymbol{\Psi}$. The part of covariance matrix $\boldsymbol{\Sigma}$ corresponding to parameters β_j incorporates the AR(1) correlation structure.

From [2] we obtain the estimator of the fixed effect in a trivariate linear mixed model, given the hyperparameters:

$$\hat{\boldsymbol{\theta}}_j = \left(\mathbf{W}^{\mathrm{T}}_{i,j} \mathbf{V}^{-1}_j \mathbf{W}_{i,j} + \boldsymbol{\Sigma}^{-1} \frac{\sigma^2_{\varepsilon,j}}{\sigma^2_{\theta,j}} \right)^{-1} \mathbf{W}^{\mathrm{T}}_{i,j} \mathbf{V}^{-1}_j \mathbf{Y}_{*,j,*},$$

where

$$\mathbf{V}^{-1}_j = \left(\tilde{\mathbf{Z}}_t \boldsymbol{\Psi} \tilde{\mathbf{Z}}^{\mathrm{T}}_t \frac{\sigma^2_{\varepsilon,j}}{\sigma^2_{\gamma,j}} + \mathbf{I} \right)^{-1}.$$

Using $\hat{\boldsymbol{\theta}}$ and previously estimated hyperparameters, the random effect estimator from a trivariate linear mixed model is:

$$\hat{\boldsymbol{\gamma}}_j(\hat{\boldsymbol{\theta}}_j) = \left(\tilde{\mathbf{Z}}^{\mathrm{T}}_t \tilde{\mathbf{Z}}_t + \boldsymbol{\Psi}^{-1} \frac{\sigma^2_{\varepsilon,j}}{\sigma^2_{\gamma,j}} \right)^{-1} \tilde{\mathbf{Z}}^{\mathrm{T}}_t \left(\mathbf{Y}_{*,j,*} - \mathbf{W}_{i,j} \hat{\boldsymbol{\theta}}_j \right).$$

Full derivation of these estimators can be found in Chapter 6 of [2].

With these analytic estimators we obtain the trivariate fit of the model. From this trivariate fit, only the parameter estimate of the middle gene in the triplet is preserved and considered its final parameter estimate. Sliding the triplet window over the genome yields final parameter estimates for each gene.

5 Optimal number of knots for penalized splines

DIC is a measure that combines model fit with model complexity (the latter is represented by the number of effective parameters) ([3]). When denoting by $\boldsymbol{\theta}_j$ the vector containing all parameters of Model (2), then the goodness-of-fit (for gene j) may measured by the deviance $D(\boldsymbol{\theta}_j) = -2\log[L(Y_{*,j,*}|\boldsymbol{\theta}_j)]$. Write $D(\bar{\boldsymbol{\theta}}_j) = D(E_{\boldsymbol{\theta}_j|Y_{*,j,*}}[\boldsymbol{\theta}_j])$ for the deviance of the posterior mean, where $\bar{\boldsymbol{\theta}}_j$ is the expectation of $\boldsymbol{\theta}_j$ and $\bar{D}(\boldsymbol{\theta}_j) = E_{\boldsymbol{\theta}_j|Y_{*,j,*}}[D(\boldsymbol{\theta}_j)]$ for the posterior mean of the deviance. The effective number of parameters $p_{D,j}$ is then calculated per gene as:

$$p_{D,j} = \bar{D}(\boldsymbol{\theta}_j) - D(\bar{\boldsymbol{\theta}}_j),$$

The effective number of parameters $p_{D,j}$ may also be defined directly through the effective degrees of freedom. For the normal hierarchical model, $p_{D,j} = \mathrm{tr}(\mathbf{H}_{\lambda,j})$, with $\mathbf{H}_{\lambda,j}$ the hat matrix ([3]). The DIC is now defined as:

$$DIC_j = D(\bar{\boldsymbol{\theta}}_j) + 2p_{D,j} = \bar{D} + p_{Dj}.$$

To decide on the optimal number of knots, we determine the DIC per gene for different numbers of knots $k = 2, \ldots, 5$. The value of k with the smallest DIC represents the model which is the best supported by the data (for that particular gene). The optimal number of knots for the whole data set (i.e. all genes) is that k which is the most frequent optimal k over the genes. For the data set from Section 2.1, Figure 4 indicates that $k = 2$ is the optimal number of knots.

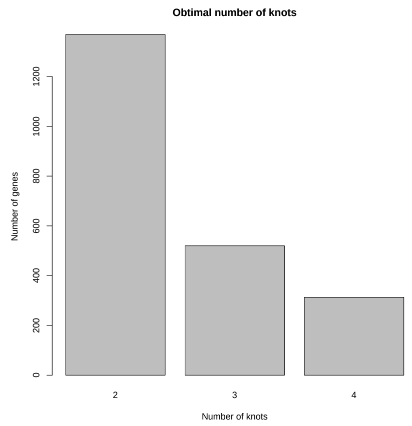

Figure 4: The bar plot illustrates the frequency of genes where the number of knots k is identified with the smallest DIC.

6 Comparison using the data set from the EDGE package

The proposed method for temporal differential expression was developed with the data set from Section 2.1 in mind. This may accidentally favor our method in the comparison with its competitors EDGE, timecourse, BATS and the reference (univariate fitted) method. Therefore the comparison is repeated using the data set from EDGE-package ([4]), thus possibly favoring the EDGE. Again sensitivity, specificity and reproducibility are compared among packages EDGE, timecourse, tigaR, BATS and the reference method. Figure 5 shows the number of true and false positive rates of the four methods. Qualitatively the same picture emerges as from the comparison using the data from Section 2.1. Similarly, when comparing the reproducibility of the methods (confer Figure 6), again tigaR and BATS outperform timecourse and EDGE.

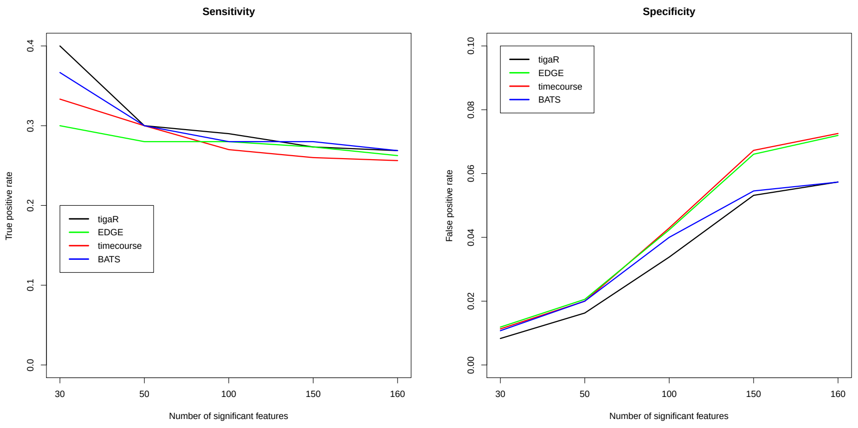

Figure 5: Plots represent comparison of tigaR, EDGE, timecourse and BATS package for the sensitivity and specificity on the data set from the EDGE-package. The left panel represents the sensitivity (of each model), where the true positive rate is plotted against the number of significant genes. The right panel illustrates the specificity (of each method), where the false positive rate is plotted against the number of significant genes.

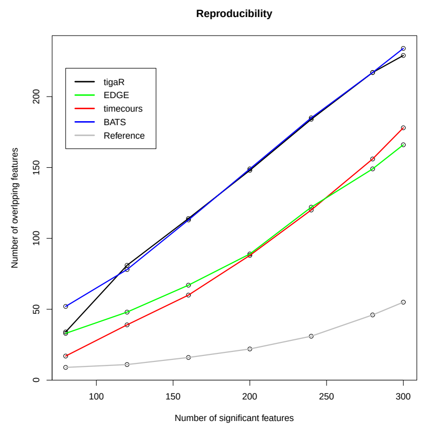

Figure 6: The plot illustrates the comparison of the reproducibility between tigaR, EDGE, timecourse, BATS and reference model. The number of significant genes is plotted against number of overlapping genes identified between two groups.

7 Additional plots

In the main manuscript Figure 2, Figure 6 and Figure 7 show only a single cell line. This is done for reasons of clarity: to make a certain effect more visible. Here, in Figure 7, Figure 8 and Figure 9 these effects are shown in all four cell lines.

Additionally, a Venn diagram (Figure 10) is presented. It illustrates the various intersections of the sets of features exhibiting temporal differential expression as declared by tigaR, EDGE, timecourse and BATS. The timecourse packages only provides a rank-ordered list of genes. For the comparison the top genes from this list are selected, their number being equal to the number of features selected by tigaR (with a common spline).

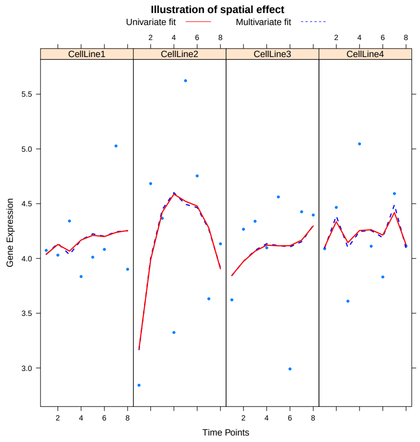

Figure 7: Illustration of the effect of the spatial prior of one gene in four cell lines. Gene expression is plotted against time (in four cell lines). The lines represent the univariate (red, solid line) and multivariate fit (blue, dashed line).

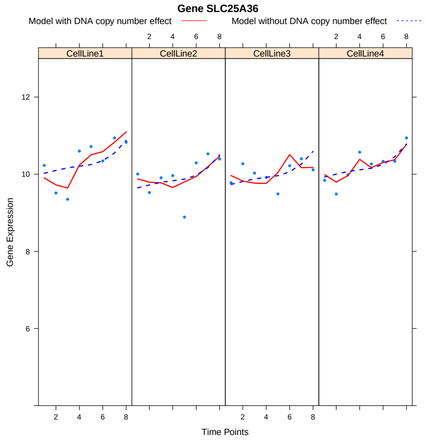

Figure 8: Dots represent expression levels of gene SLC25A36 plotted against time in four cell lines. The solid (red) line represents the full model while the dashed (blue) line illustrates the fit of the model without copy number effect.

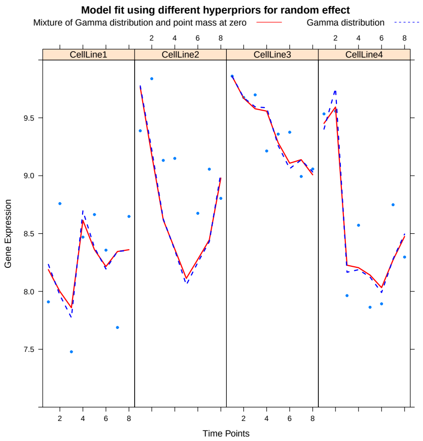

Figure 9: Effect of using different priors in four cell lines. Gene expression is plotted against time (in four cell lines). The solid red line is the fit of the model with a standard prior, while the dashed blue line is that of the model with an alternative prior.

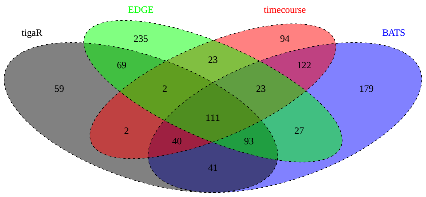

Figure 10: Venn diagram of methods tigaR, EDGE, timecourse and BATS.

Author details

References

1. Hastie, T.J., Tibshirani, R.J.: Generalized Additive Models. CRC Press, London (1990)
2. Sorensen, D., Gianola, D.: Likelihood, Bayesian and MCMC Methods in Quantitative Genetics. Springer, New York (2002)
3. Spiegelhalter, D.J., Best, N.G., Carlin, B.P., van der Liner, A.: Bayesian measures of model complexity and fit. Journal of the Royal Statistical Society: Series B (Statistical Methodology) **64**, 583–639 (2002)
4. Storey, J.D., Xiao, W., Leek, J.T., Tompkins, R.G., Davis, R.W.: Significance analysis of time course microarray experiments. Proceedings of the National Academy of Sciences of the United States of America **102**, 12837–12842 (2005)
